# Identification of a Circulating MicroRNA Signature for Colorectal Cancer Detection

**DOI:** 10.1371/journal.pone.0087451

**Published:** 2014-04-07

**Authors:** Jia Wang, Sheng-kai Huang, Mei Zhao, Mei Yang, Jia-ling Zhong, Yu-yu Gu, Hua Peng, Yi-qun Che, Chang-zhi Huang

**Affiliations:** 1 Department of Etiology and Carcinogenesis, State Key Laboratory of Molecular Oncology, Cancer Institute and Hospital, Chinese Academy of Medical Sciences and Peking Union Medical College, Beijing, China; 2 Department of Clinical Laboratory, Cancer Institute & Hospital, Chinese Academy of Medical Sciences & Peking Union Medical College, Beijing, China; IPMC, CNRS UMR 7275 UNS, France

## Abstract

Prognosis of patients with colorectal cancer (CRC) is generally poor because of the lack of simple, convenient, and noninvasive tools for CRC detection at the early stage. The discovery of microRNAs (miRNAs) and their different expression profiles among different kinds of diseases has opened a new avenue for tumor diagnosis. We built a serum microRNA expression profile signature and tested its specificity and sensitivity as a biomarker in the diagnosis of CRC. We also studied its possible role in monitoring the progression of CRC. We conducted a two phase case-control test to identify serum miRNAs as biomarkers for CRC diagnosis. Using quantitative reverse transcription polymerase chain reactions, we tested ten candidate miRNAs in a training set (30 CRCs vs 30 controls). Risk score analysis was used to evaluate the diagnostic value of the serum miRNA profiling system. Other independent samples, including 83 CRCs and 59 controls, were used to validate the diagnostic model. In the training set, six serum miRNAs (miR-21, let-7g, miR-31, miR-92a, miR-181b, and miR-203) had significantly different expression levels between the CRCs and healthy controls. Risk score analysis demonstrated that the six-miRNA-based biomarker signature had high sensitivity and specificity for distinguishing the CRC samples from cancer-free controls. The areas under the receiver operating characteristic (ROC) curve of the six-miRNA signature profiles were 0.900 and 0.923 for the two sets of serum samples, respectively. However, for the same serum samples, the areas under the ROC curve used by the tumor markers carcinoembryonic antigen (CEA) and carbohydrate antigen 19-9 (CA19-9) were only 0.649 and 0.598, respectively. The expression levels of the six serum miRNAs were also correlated with CRC progression. Thus, the identified six-miRNA signature can be used as a noninvasive biomarker for the diagnosis of CRC, with relatively high sensitivity and specificity.

## Introduction

Colorectal cancer (CRC) is the third most common cancer in the world. It accounts for nearly 50,000 deaths each year and is the second leading cause of cancer-related death [1], [2]. It was estimated that, every year, one and half million new CRC cases would be diagnosed worldwide [2]. A study registered in the National Cancer Institute's Surveillance Epidemiology and End Results (SEER) database, was conducted with 119,363 people diagnosed with colon adenocarcinoma between 1991 and 2000. This study found that the observed 5-year survival rates were related to the stage of the disease at diagnosis; for patients diagnosed in I/IIa stage the survival was much better than for patients diagnosed in later stages [3]. Although qualified care and screening programs play important roles in the survival of patients with CRC, surgical resection in the early stage is the most effective treatment and prolongs the survival of patients. Unfortunately, early-stage CRCs are difficult to detect because of fewer symptoms.

Currently, endoscopy and fecal occult blood tests (FOBT) are often used in clinics to diagnose CRC patients. However, not only is random biopsy an invasive procedure, but potential sampling errors may occur, which further limits their efficacy. Meanwhile, although FOBT is simple, inexpensive and noninvasive, it presents particular poor sensitivity for the detection of early-stage CRC [4], [5]. The proteome of circulating blood has also been applied to detect biomarkers for CRC such as carcinoembryonic antigen (CEA) and carbohydrate antigen 19-9 (CA19-9), but its sensitivity and specificity, especially for early stage colorectal cancer, seems to be insufficient [6]. Therefore, new methods and novel diagnostic biomarkers are urgently required for mass surveys of early events of CRC.

MicroRNA (miRNA) is a ∼22-nt long non-coding RNA, which plays a negative role in gene expression [7], [8]. Altered expression of miRNAs has been associated with various diseases, particularly cancer. MiRNAs have been shown to successfully differentiate diverse cancers and predict outcomes in both solid and hematological malignancies [7]. Recent studies have shown that there are large amounts of miRNAs in the circulation. These circulating miRNAs are able to withstand unfavorable physiological conditions, such as extreme variations in pH, temperature, and multiple freeze/thaw cycles [9], [10]. Furthermore, some researchers pointed out that the profiles of circulating miRNA showed consistent expression levels across physiologically healthy individuals [11]. Because serum and plasma are relatively easy to access, circulating miRNA is one of the most promising candidates for the diagnosis of cancer. Many studies have shown that the expression patterns of serum miRNAs can potentially identify various types of cancer, including lung cancer, prostate cancer, breast cancer, ovarian cancer, and liver cancer [12], [13]. Therefore, identifying a unique serum miRNA expression profile for CRC will be useful in the diagnosis and follow-up treatment of the tumors.

To strengthen the diagnostic efficiency of circulation miRNAs, new methods to elevate their diagnostic value for CRC are required. Many researchers found that the combination of several miRNAs as a biomarker could improve the diagnostic efficiency [14], [15]. However, these studies focused mainly on up-regulated miRNAs. In this study, we aimed to combine both up- and down-regulated miRNAs, to build a diagnostic model for CRC. We first validated ten miRNAs previously reported to be associated with CRC, namely miR-21, miR-31, miR-203, miR-92a, miR-181b, miR-145, miR-143, miR-30c, miR-17, and let-7g. Then, by statistical analysis, we identified a profile that combined six serum miRNAs, which can serve as a novel noninvasive biomarker for CRC diagnosis.

## Materials and Methods

### Ethics statement

Written informed consent was obtained from all patients and healthy volunteers before the study, and all samples were collected according to the protocols approved by the Clinical Research Ethics Committee of the Cancer Institute and Hospital, Chinese Academy of Medical Sciences.

### Study design and patients

**Figure 1:**
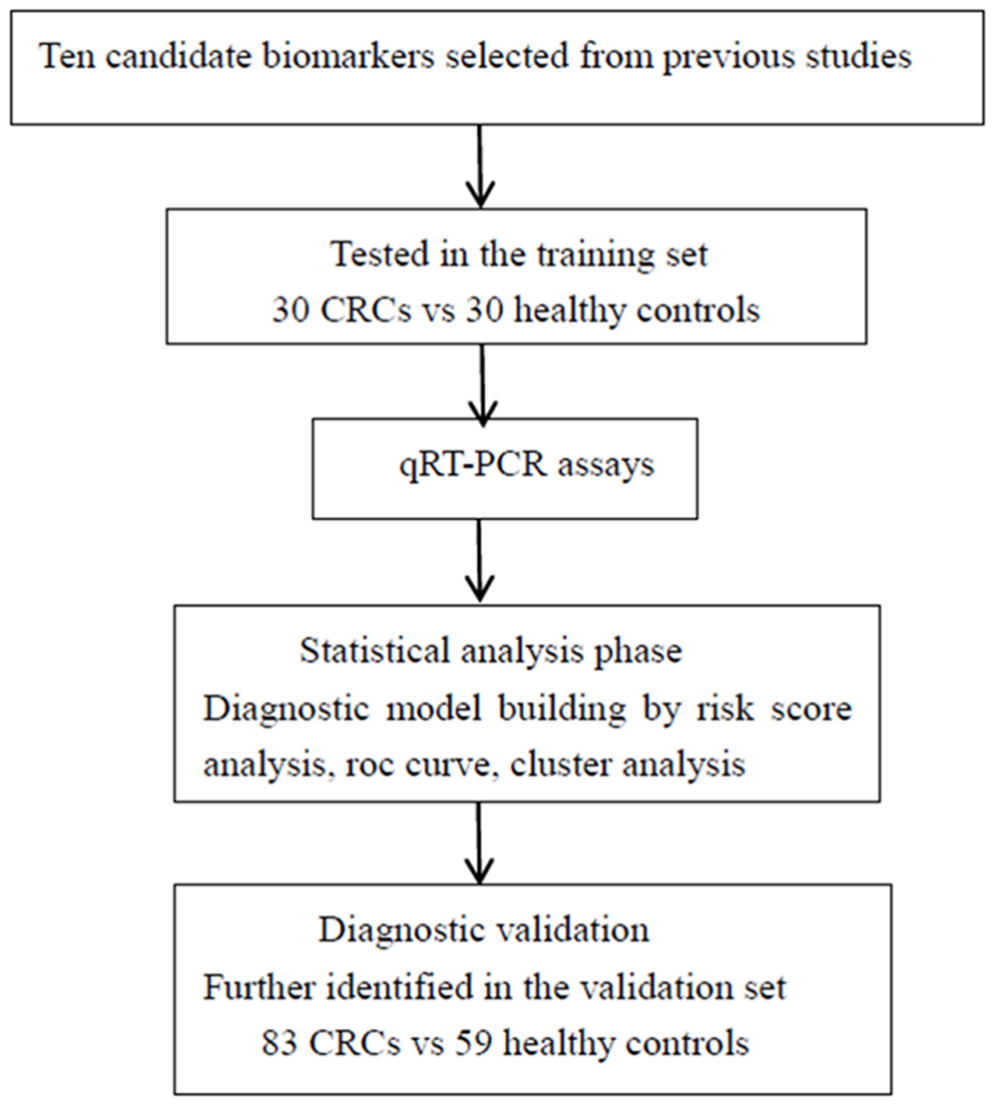
Overview of the design strategy used in this study.

To identify a surrogate biomarker for CRC, a multi-stage case–control study was designed to identify a diagnostic serum miRNA profile (see Figure 1). A total of 113 patients with primary CRCs (Stages I–IV) and 89 control subjects were recruited for this study. All 113 cancer patients were recruited from the Cancer Institute and Hospital, Chinese Academy of Medical Sciences between 2008 and 2010. Patients with a previous history of malignant tumors, hereditary non-polyposis CRC, or familial adenomatous polyposis, were excluded. Serum samples were collected prior to any tumor resection such as surgery, chemotherapy, and/or radiotherapy. Each patient had a histologically confirmed diagnosis, and the tumor stage was determined based mainly on surgery findings, or on biopsy and imaging technology when the tumor was not suitable for surgical treatment. Healthy controls with no known malignancy or active inflammatory condition were matched to the patients based on age, gender, and ethnicity. The TNM classification system was used to assess the tumor stage according to the tumor-node-metastasis staging system of the Sixth Edition of the American Joint Commission. The demographics and clinical features of the study cohort are listed in Table 1.

**Table 1:** Demographic and clinical features of the colorectal cancer (CRC) patients and healthy controls.

Characteristics		Healthy controls n = 89	CRC cases n = 113	p-value
Age (year, Mean ± SD)		57±10.4	55±7.6	p = 0.762^a^
Gender	Male	45	65	p = 0.324^b^
	Female	44	48	
TNM stage	I		19	
	II		43	
	III		38	
	IV		13	
Family history of CRC	Yes		9	
	No		104	
Carcinoembryonic antigen (CEA)	Positive	5	40	p<0.0001^c^
	Negative	84	73	
Carbohydrate antigen 19-9 (CA19-9)	Positive	3	26	P<0.0001^c^
	Negative	86	87	
Significant cardiac dysfunction	Yes	7	12	P = 0.630^c^
	No	82	101	
Neurological disease or diabetes	Yes	1	4	P = 0.583^c^
	No	88	109	

a Student's- t test.

b Two-side chi-squared test.

c Fisher's exact test.

In the biomarker selection stage, a panel of ten candidate miRNAs (miR-21, let-7g, miR-31, miR-92a, miR-181b, miR-203, miR-17, miR-30c, miR-143, and miR-145) were selected for investigation in serum samples based on earlier reviews on colorectal cancer [13], [16]. For the training set, we randomly selected 30 CRCs and 30 healthy controls and performed quantitative reverse transcription polymerase chain reactions (qRT-PCRs) to select candidate miRNAs. Subsequently, validation was performed using the remaining 83 CRC and 59 healthy donor samples (validation set).

### Serum collection, RNA isolation, and qRT-PCR assay

Venous blood samples (≈5 ml) were collected into serum collection tubes, and left at room temperature for about 1 h before being centrifuged at 820 g for 10 min at 4°C. The resulting serum was transferred into new tubes, followed by further centrifugation at 16,000×*g* for 10 min at 4°C, to completely remove any cell debris. Total RNA was isolated from 250 µl of serum using the Trizol LS Reagent (Invitrogen, Carlsbad, CA, USA) co-purification technique. For each 250 µl of serum, phase separation was performed by the addition of 750 µl of Trizol LS. Then, 200 µl of trichloromethane was added to augment the RNA phase separation process. Total RNA was precipitated using isopropanol and washed with 75% ethanol, then 30 µl of RNase free water was added for solubilization. Each 250 µl of serum yielded 30 µl of total RNA solution, which was stored at −80°C. Three µl of total RNA was polyadenylated by poly (A) polymerase and reverse transcribed to cDNA using the TaKaRa microRNA transcription kit (Takara, Japan), following the manufacturer's protocol. The reverse transcribed products were diluted to one fifth with RNase-free water and used as templates for further PCR analysis. PCR was performed using the SYBR Premix Ex Taq II kit (Takara) in an ABI 7300 Real-Time PCR System (Applied Biosystems, Foster City, CA, USA) with the manufacturer-provided universal primer and miRNA-specific forward primer. The primer sequences are listed in Table 2. Each reaction was performed in a 20 µl volume system containing 2 µl cDNA, 0.4 µl of each primer (10 µM), 0.4 µl ROX reference dye (50×), 6.8 µl sterile distilled water, and 2× SYBR Premix Ex Taq II. The PCR program was: denaturation at 95°C for 30 second, followed by 40 cycles of denaturation for 5 s at 95°C, and extension for 31 s at 60°C. At the end of the PCR cycles, melting curve analyses were performed to validate the specificity of the expected PCR product. Each reaction was carried out in triplicate, and the cDNA dilution was used as the template for the negative control.

**Table 2:** Primers used for qRT-PCR in this study.

Primers	Sequences
miR-21	CGGCGTAGCTTATCAGACTGATG
let-7g	GCGCTGAGGTAGTAGTTTGACAG
miR-92a	TATTGCACTTGTCCCGGCCT
miR-181b	AACATTCATTGCTGTCGGTGG
miR-16	TAGCAGCACGTAAATATTGGCG
miR-31	AGGCAAGATGCTGGCATAGCT
miR-203	GCCGTGAAATGTTTAGGACCAC
miR-17	CAAAGTGCTTACAGTGCAGGTAG
miR-143	GCTGAGATGAAGCACTGTAGCTC
miR-145	GTCCAGTTTTCCCAGGAAUCCCT
miR-203	CGGCGTGTAAACATCCTACACTC

Endogenous miR-16 was used as the normalizer for circulating miRNA quantification. The relative expression levels of the miRNAs were calculated by the comparative 2-^ΔΔCT^ method as described previously [17], [18].

### Serum CA19-9 and CEA analysis

The tumor markers CEA and CA-199 were analyzed with an Elecsys immunoassay analyzer (Roche, Basil, Switzerland). The upper normal limits for the tumor markers were 6.5 ng/ml for CEA and 39 U/ml for CA-199.

### Statistical analysis

Comparison of the demographic and clinical features between the CRC patients and the healthy controls was determined by the Student's t-test, one-way analysis of variance (ANOVA), or chi-squared test.

Risk score analysis was performed to evaluate the association between the CRC samples and the miRNA expression levels. The risk score of each miRNA in the training set was denoted as *s*. For up-regulated miRNAs, the risk score was set as 1 if the expression level was higher in the CRC samples than the upper 95% reference interval for the corresponding miRNA level in the controls, and for the down-regulated miRNAs, risk score was set as −1 if the expression level was lower in the CRC samples than the lower 95% reference interval in the controls; otherwise, the score was set as 0. For the correlation of each miRNA with CRC risk, each patient was assigned a risk score function (RSF) based on a linear combination of the expression levels of the miRNAs. Using the information from six miRNAs, the RSF for sample *i* is as follows:
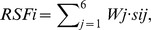
In the equation above, *sij* is the risk score for miRNA *j* on sample *i*, and W*j* is the weight of the risk score of miRNA *j*. To determine the Ws, six univariate logistic regression models were fitted with the disease status with each of the risk scores. The regression coefficient of each risk score was used as the weight to indicate the contribution of each miRNA to the RSF. The frequency table and receiver operating characteristic (ROC) curves were then used to evaluate the diagnostic effects of the profiling and to find an appropriate cutoff point. Validation of the procedure and the cutoffs were performed on the validation set.

An independent samples t-test was used to compare serum miRNA concentrations between the cancer and healthy samples. Because of the magnitude and range of relative miRNA expression levels that were observed, the results data were log-transformed for analysis (log_2_). All tests were 2-sided and a significance level of p-value<0.05 was considered statistically significant. All the statistical analyses were performed using SPSS 16.0 software (SPSS Inc., Chicago, IL, USA) and graphs were generated with Graphpad Prism 5.0 (Graphpad Software Inc., San Diego, CA, USA). Data are presented as the mean. For the cluster analysis, hierarchical clustering was performed using Cluster 3.0 (Berkeley, CA, USA) with the complete linkage method.

## Results

### Description of the patients

All the 113 patients enrolled in the present study had clinical and pathological diagnosis of CRC. As shown in Table 1, there was no significant difference in the distribution of age, gender and other diseases, such as cardiac dysfunction or neurological disease, between the CRC patients and the healthy controls in the training and validation sets. Elevated levels of CEA (>6.5 ng/ml) and CA19-9 (>39 U/ml) were found in 40 and 26 patients, respectively.

### Evaluation of miRNA expression in CRC patients and healthy controls by real-time qRT-PCR analysis

**Figure 2:**
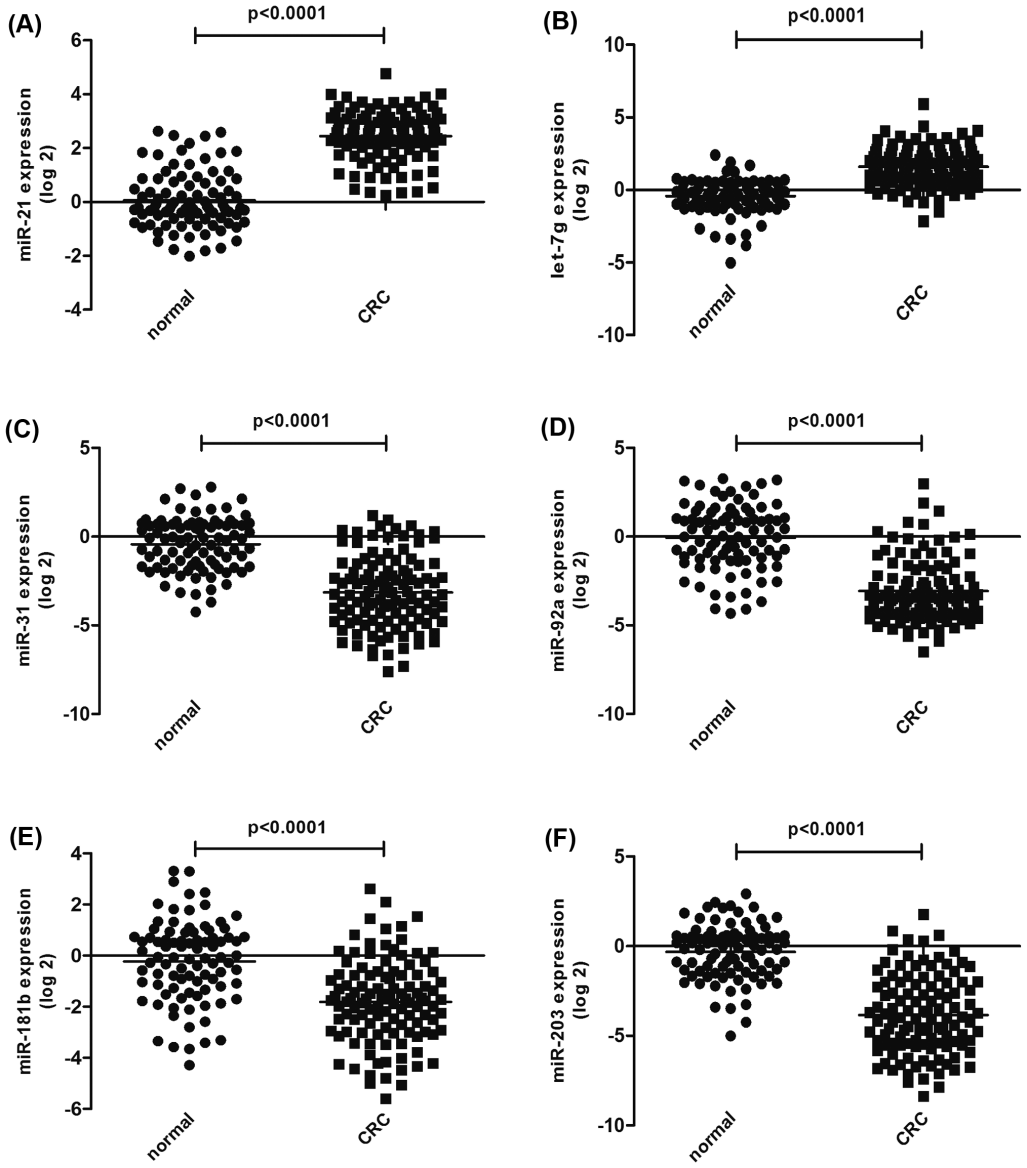
Different expression levels of six selected miRNAs in the CRC and healthy control serum samples. The serum expression levels of the six selected miRNAs were measured in 113 CRC cases and 89 healthy control subjects (in both the training set and the validation set) using a SYBR-based qRT-PCR assay. Each reaction was carried out in triplicate.

A two phase case-control test was designed to identify serum miRNAs as candidate biomarkers for CRC diagnosis. We selected ten candidate miRNAs reported in previous studies, and used qRT-PCR assays to confirm their expression in the 30 CRC cases and 30 age- and gender-matched healthy controls in the training set. MiR-16 expression was used to normalize the qRT-PCR data. No significant difference was observed in the levels of miR-17, miR-145, miR-143, miR-30c between the CRC and control samples (data not shown). However, the expression levels of six of the miRNAs were significantly different between the CRC and control samples; two miRNAs, miR-21 and let-7g, were up-regulated, and four miRNAs, miR-31, miR-92a, miR-181b, and miR-203, were down-regulated (Table 3). The concentrations of the six differentially expressed miRNAs were examined by qRT-PCR in the 83 CRC patients and 59 matched controls in the validation set. The alterations in the miRNA expression patterns observed in the validation set were consistent with those found in the training set. The differential expression of the six miRNAs in the 113 CRC cases compared to the 89 controls is shown in Figure 2. Thus, this two-phase test and analysis process generated a profile of six serum miRNAs that served as a potential biomarker for CRC. The six candidate biomarkers were then subjected to further tests and analyses.

**Table 3:** Differentially expressed miRNAs in CRC serum samples compared with serum samples from the controls in the training and validation sets.

miRNA	Training set (30 vs 30)		Average fold change	P-value	Validation set (59 vs 83)		Average fold change	p-value
	Controls	CRCs			Controls	CRCs		
miR-21	1.42±1.43	5.51±5.64	3.88	P<0.0001	1.39±1.26	6.46±3.12	4.65	p<0.0001
Let-7g	0.99±0.82	4.16±2.85	4.20	P<0.0001	0.98±0.76	4.93±7.44	5.03	p<0.0001
miR-31	1.66±1.69	0.38±0.53	0.23	P<0.0001	1.41±2.24	0.24±0.36	0.17	p<0.0001
miR-92a	1.76±1.66	0.47±0.75	0.27	P<0.0001	1.74±2.04	0.20±0.30	0.11	p<0.0001
miR-181b	1.28±0.85	0.50±0.85	0.39	P = 0.001	1.75±2.66	0.57±0.83	0.33	p<0.0001
miR-203	1.43±1.15	0.29±0.67	0.20	P<0.0001	1.45±2.13	0.19±0.33	0.13	p<0.0001

The normalized miRNAs expression levels are presented as mean±SD.

### Separation of CRC cases and healthy controls by risk score analysis

**Figure 3:**
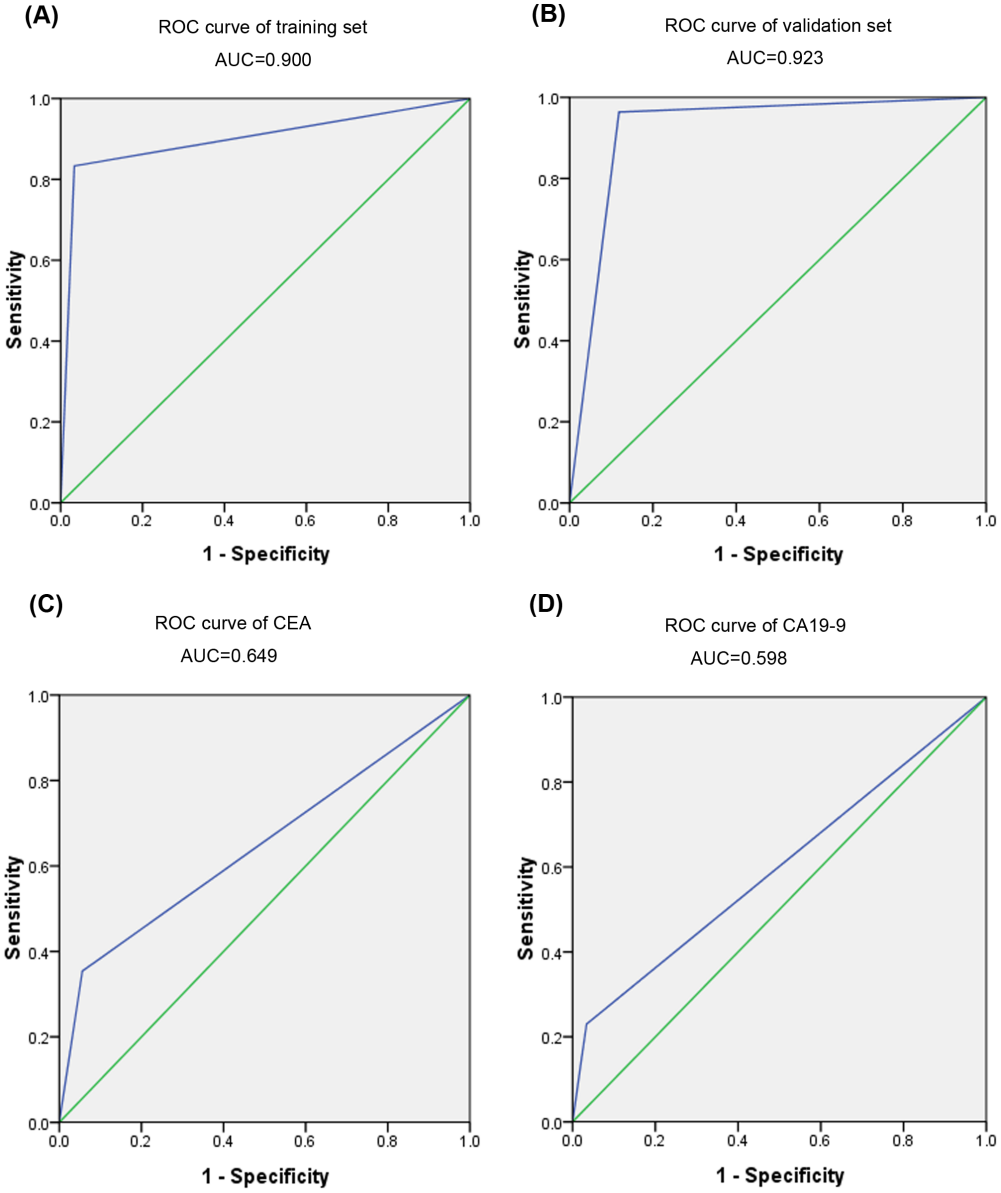
Sensitivity and specificity of the six-miRNA signature for discriminating between CRC and healthy control samples. (A) ROC curve analysis for the profile of the six- miRNA signature in the training set yielded an AUC value of 0.900 (95% CI: 0.812–0.988) with 83.3% sensitivity and 96.7% specificity (cut-off value = 9.595). (B) ROC curve analysis for the profile of the six-miRNA signature in the validation set yielded an AUC value of 0.923 (95% CI: 0.869–0.976) with 96.4% sensitivity and 88.1% specificity (cut-off value = 9.595). (C) Carcinoembryonic antigen (CEA) yielded an AUC value of 0.649 (95% CI): 0.574–0.724) with 35.4% sensitivity and 94.4% specificity and (D) carbohydrate antigen 19-9 (CA19-9) yielded an AUC value of 0.598 (95% CI: 0.521–0.676) with 23% sensitivity and 96.6% specificity, for the same serum samples.

To evaluate the diagnostic value of a miRNA expression signature comprising the six identified miRNAs, we performed a risk score analysis to distinguish between the CRC serum samples and the serum samples from the healthy controls. First, the risk score for each of the miRNAs in the training set was calculated using the risk score formula. At the optimal cutoff predictive value of 9.595 for the risk score function, when the sensitivity and specificity were at their maximum, the serum samples were divided into a low-risk group and a high-risk group representing the healthy donors and CRC cases, respectively. Next, we used the cutoff value to analysis the 142 samples in the validation set. As shown in Table 4, the positive predictive value and negative predictive value of the six-miRNA signature in the training set were 0.96 and 0.85 respectively, and in the validation set the positive and negative predictive values were 0.95 and 0.92 respectively. We also constructed ROC curves to estimate the sensitivity and specificity of the miRNA-based biomarkers. The areas under the curves (AUCs) were 0.900 and 0.923 for the training set and validation set, respectively (Figure 3A and 3B). Using the same serum samples, we compared the AUCs for the six-miRNA signature with the AUCs for the tumor markers CEA and CA19-9, which are currently used in clinics for CRC detection. The AUC values for the six-miRNA signature were markedly higher than the AUCs for CEA (0.649) and CA19-9 (0.598) (Figure 3C and 3D). These results indicate that the six-miRNA signature is a more accurate biomarker than CEA and CA19-9 for CRC diagnosis.

**Table 4:** Risk score analysis of CRC patients and healthy controls.

	Score	<9.595	>9.595	PPV^a^	NPV^b^
Training set	Control	29	1	0.96	0.85
	CRC	5	25		
Validation set	Control	52	7	0.95	0.92
	CRC	3	80		

a Positive predictive value.

b Negative predictive value.

### Association with demographic and clinical factors

To determine the role of the six miRNA-based tumor markers in CRC development, the CRC cases were further stratified based on their TNM staging. In this analysis, the CRC samples from training set and validation set were combined and the expression levels of the six miRNAs were correlated with the tumor stage of the CRC patients. We found that the risk score values were different among CRC cases at different tumor stages (see Figure S1A). The mean risk score of CRC cases at later stages (IIb, III, and IV) was significantly higher than the score at earlier stages (I and IIa). There was no significant association between the six miRNAs and gender, age, nodal status, and tumor invasive depth (see Figure S1B–E).

### Unsupervised cluster analysis

To analyze the differential expression of the miRNAs between the CRC and control serum samples, we conducted an unsupervised clustering process that was blind to the clinical annotations. The dendrogram showed a clear separation of the CRC samples from the control samples based on the six- miRNA signature profile (see Figure S2). In the training set, none of 30 CRC samples and only four of 30 control samples were incorrectly classified (Figure S2A). In the validation set, the 83 CRC cases and 59 controls were classified into two main categories; only one CRC case and three controls were misclassified (Figure S2B).

## Discussion

Many studies have found that miRNA expression is aberrant in CRC development; however, most of these studies focused on the expression of miRNAs in tumor tissues and cells. Although tissue miRNAs can provide an accurate diagnosis for various types of cancer, the difficulty in collecting tissue samples limits its application for the detection of cancer biomarkers. Acquiring tissue samples is an invasive procedure and depends on surgical sections after initial clinical classification. The search for noninvasive tools for the diagnosis of cancer has long been a goal of many researchers, and much of the interest has been on the circulation of nucleic acids in plasma and serum. Compared with DNA and mRNA, circulating miRNAs show remarkable stability after prolonged incubation at room temperature and/or multiple freezing-thawing processes. However, the protective mechanism of circulating miRNAs is still unknown. Some investigators reported that circulating miRNAs were in the form of argonaute 2 (Ago2)-miRNA complexes that could avoid RNase digestion [19]. MiRNAs in plasma can be secreted from cells and the release process may be a selective mechanism that is correlated with malignancy [20]. There are many advantages in using circulating miRNAs for CRC detection. Sample collection is easy and low cost, and importantly, is a relatively noninvasive procedure that can be applied easily in the screening and monitoring of cancer patients. Furthermore, miRNAs profiles are mainly consistent among healthy individuals and are very stable in the blood.

Early searches for noninvasive tools for the diagnosis of cancers were focused mainly on single or only a few tumor-specific miRNAs [9], [21]–[23]. For example, Liu et al. [21] studied the miRNA profiles in the serum of gastric cancer patients, colorectal cancer patients, and healthy individuals. Interestingly, they found that circulating miR-378 was related to gastric cancer, but was not associated with other gastrointestinal cancers. They reported that miR-378 expression levels could distinguish gastric cancer patients from healthy controls, with 87.5% sensitivity and 70.73% specificity [21]. Serum miR-1246 alone yielded an area under the ROC curve of 0.754, with 71.3% sensitivity and 73.9% specificity for distinguishing esophageal cancer patients from healthy controls [23]. Although this kind of approach is simple and available, the specificity of biomarkers based on a single tumor-specific miRNA is generally poor. The initiation and development of cancers involve many diverse and complex molecular events, which will affect the proliferation, cell cycles, and apoptosis. Therefore, a combination of multiple serum miRNAs should be more reliable for tumor detection than the conventional single protein-based or carbohydrate-based biomarkers. In this study, we identified two up-regulated serum miRNAs (miR-21, let-7g), and four down-regulated miRNAs (miR-31, miR-181b, miR-92a, miR-203). To the best of our knowledge, this is the first study to describe a serum miRNA-based signature that was built by combining over- and under-expressed miRNAs, both of which play significant roles in the tumor proliferation, migration, and invasion. The six-miRNA signature could detect CRC serum samples with a sensitivity and specificity of 93% and 91%, respectively (see Table S2), significantly higher than any single-factor biomarker, such as CA19-9 or CEA. Our results showed that, for the same serum samples, the sensitivity of CRC detection by CA19-9 and CEA were 35% and 23%, respectively. Clearly, the combined six-miRNA signature can successfully separate the CRC patients from the controls, and could serve as an accurate biomarker for CRC diagnosis. In particular, this biomarker showed a different expression profile between the normal controls and CRC patients with only stage I/II cancers (Table S1). CRC patients with stage I/II can undergo complete resection of the tumor, and will have a better prognosis. Our data strongly suggest that the application of the six-miRNA signature as a biomarker for defining early-stage CRC can be an effective way to change outcomes and improve prognosis. We have found that miRNA expression is correlated with tumor stage, and the serum miRNA signature can be used to detect the progression stage of CRC. No differences were found when the CRC cases were stratified by demographic and clinical factor, such as gender, age, nodal status, and tumor invasive depth; however, our results showed that a high risk score was associated with the advanced clinical stages of this disease (Figure S1). Currently, TNM staging system is the main tool used by clinicians to estimate tumor burden and the predict prognosis and survival. We propose that the six-miRNA signature in serum samples could become an important predictive parameter in choosing the best combination of treatment modalities, such as surgery, radiation, and/or chemotherapy. However, our data are partly contradictory with the results of some previous studies; for example, the expression levels of miR-181b, miR-92a, miR-31, and miR-203 were reported to be elevated in CRC tissue samples compared with controls. This difference may be attributed to different miRNA expression levels between tissue and plasma. Many studies have shown that miRNA expression in tissue samples changed in the opposite direction from their expression in serum samples [21], [24]. This may be caused by the cellular selection mechanism of miRNA release [20]; for example, cancer cells may selectively retain some miRNAs, resulting in a decrease of miRNAs in serum. More interestingly, the expression of these four down-regulated miRNAs was also found to be down-regulated in some other types of cancer. For example, miR-31, which is located on chromosome 9p21.3, was significantly lower in bladder cancer [25], breast cancer [26], gastric cancer [27], prostate carcinoma [28], and CRC with brain metastasis [29]. Many researchers have found that miR-31 can inhibit breast cancer metastasis [26], [30], and deregulation of miR-31 has been associated with cancer progression and metastasis. The high expression of miR-92a has been associated with the development and progression of many cancers [31]; however, Shigoka et al. [32] found that miR-92a was highly expressed in hepatocellular carcinoma tissue, was lower in the plasma of these patients, but was elevated in their plasma after surgical treatment. Nilsson et al. [33] showed that low miR-92a levels in tumors were associated with the stage of the tumor and that its down-regulation increased cell migration. MiR-181b has been shown to act as a tumor suppressor gene, and was down-regulated in human gliomas [34], [35].

In summary, this is the first study to report the clinically diagnostic value of a six-miRNA-based biomarker signature in serum that contains both up- and down-regulated miRNAs. Our work will serve as a basis for further investigation, preferably large-scale validation in clinical trials, before serum miRNAs can be used as a routine screening tool for CRC.

## Supporting Information

Figure S1 **The association of expression levels of the six miRNAs with demographic and clinical factors of CRC patients.** (A) When CRC cases were grouped by their TNM staging, the mean risk score of the CRC cases at later stages (IIb, III, and IV) was significantly higher than at earlier stages (I and IIa) (p<0.05). (B–E) There was no significant association between the six miRNAs and tumor invasive depth, nodal status, gender and age.
(DOCX)

Figure S2 **Dendrogram of the unsupervised clustering results.** The dendrogram indicates a clear separation of the CRC samples from the control samples based on the six-miRNA signature in both the training set (A) and the validation set (B).
(DOCX)

Table S1 **Differentially-expressed miRNAs in stage I/II CRC serum samples compared with in control serum samples.** The normalized miRNAs expression levels are presented as mean ± SD.
(DOCX)

Table S2 **Sensitivity and specificity of the six-miRNA biomarker signature compared with the CEA and CA19-9 markers.**
(DOCX)
