# Supplementary material for: Identification of a Circulating MicroRNA Signature for Colorectal Cancer Detection
Source: PLoS One. 2014 Apr 7;9(4):e87451. doi: 10.1371/journal.pone.0087451 (PMC3977854; doi:10.1371/journal.pone.0087451)
Supplement: Figure S1 — The association of expression levels of the six miRNAs with demographic and clinical factors of CRC patients. (A) When CRC cases were grouped by their TNM staging, the mean risk score of the CRC cases at later stages (IIb, III, and IV) was significantly higher than at earlier stages (I and IIa) (p<0.05). (B–E) There was no significant association between the six miRNAs and tumor invasive depth, nodal status, gender and age. (DOCX) [file pone.0087451.s001.docx]

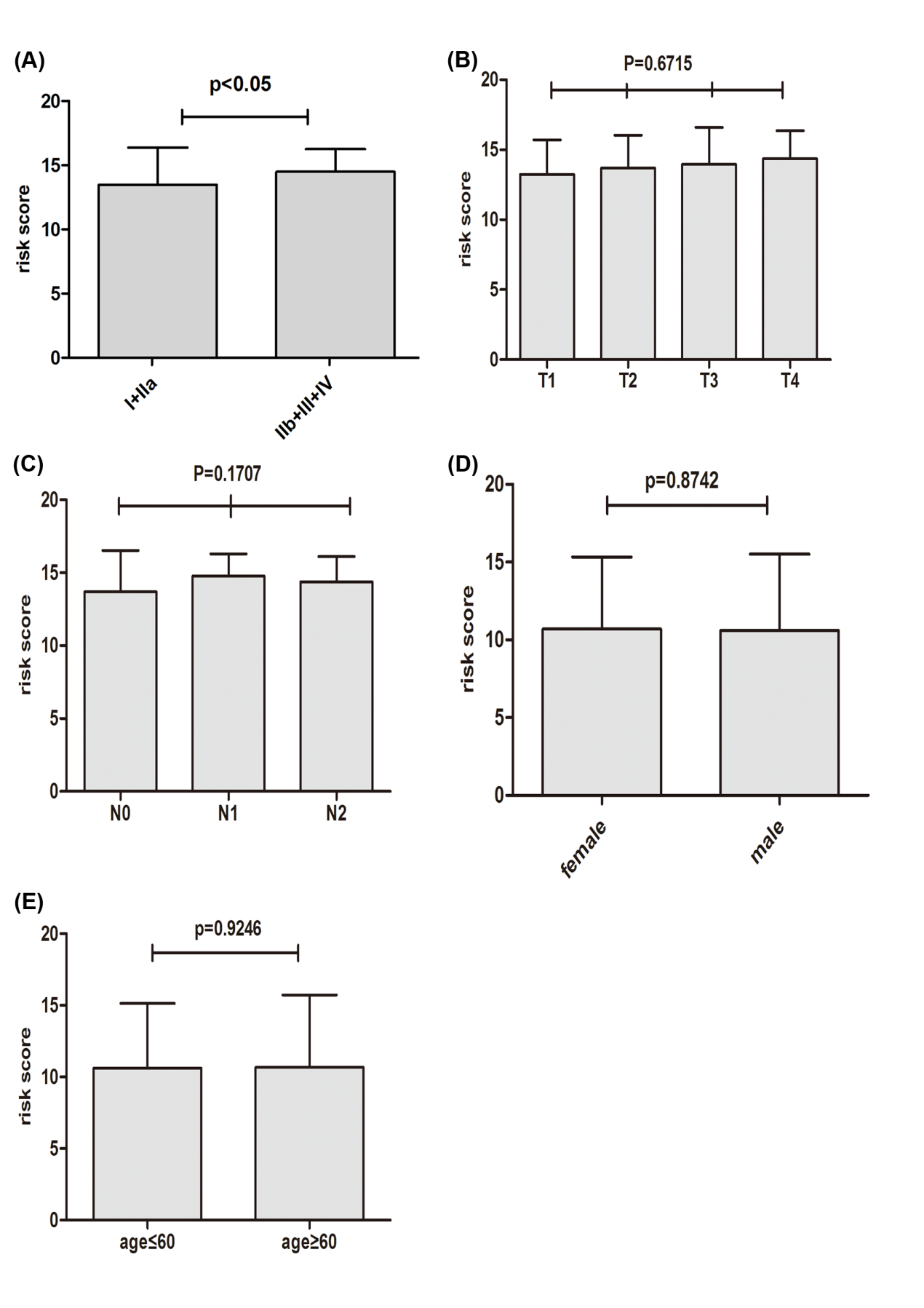


**Figure S1. The association of expression levels of the six miRNAs with demographic and clinical factors of CRC patients.** (A) When CRC cases were grouped by their TNM staging, the mean risk score of the CRC cases at later stages (IIb, III, and IV) was significantly higher than at earlier stages (I and IIa) (p <0.05). (B-E) There was no significant association between the six miRNAs and tumor invasive depth, nodal status, gender and age.
