# Supplementary material for: Identification of a Circulating MicroRNA Signature for Colorectal Cancer Detection
Source: PLoS One. 2014 Apr 7;9(4):e87451. doi: 10.1371/journal.pone.0087451 (PMC3977854; doi:10.1371/journal.pone.0087451)
Supplement: Figure S2 — Dendrogram of the unsupervised clustering results. The dendrogram indicates a clear separation of the CRC samples from the control samples based on the six-miRNA signature in both the training set (A) and the validation set (B). (DOCX) [file pone.0087451.s002.docx]

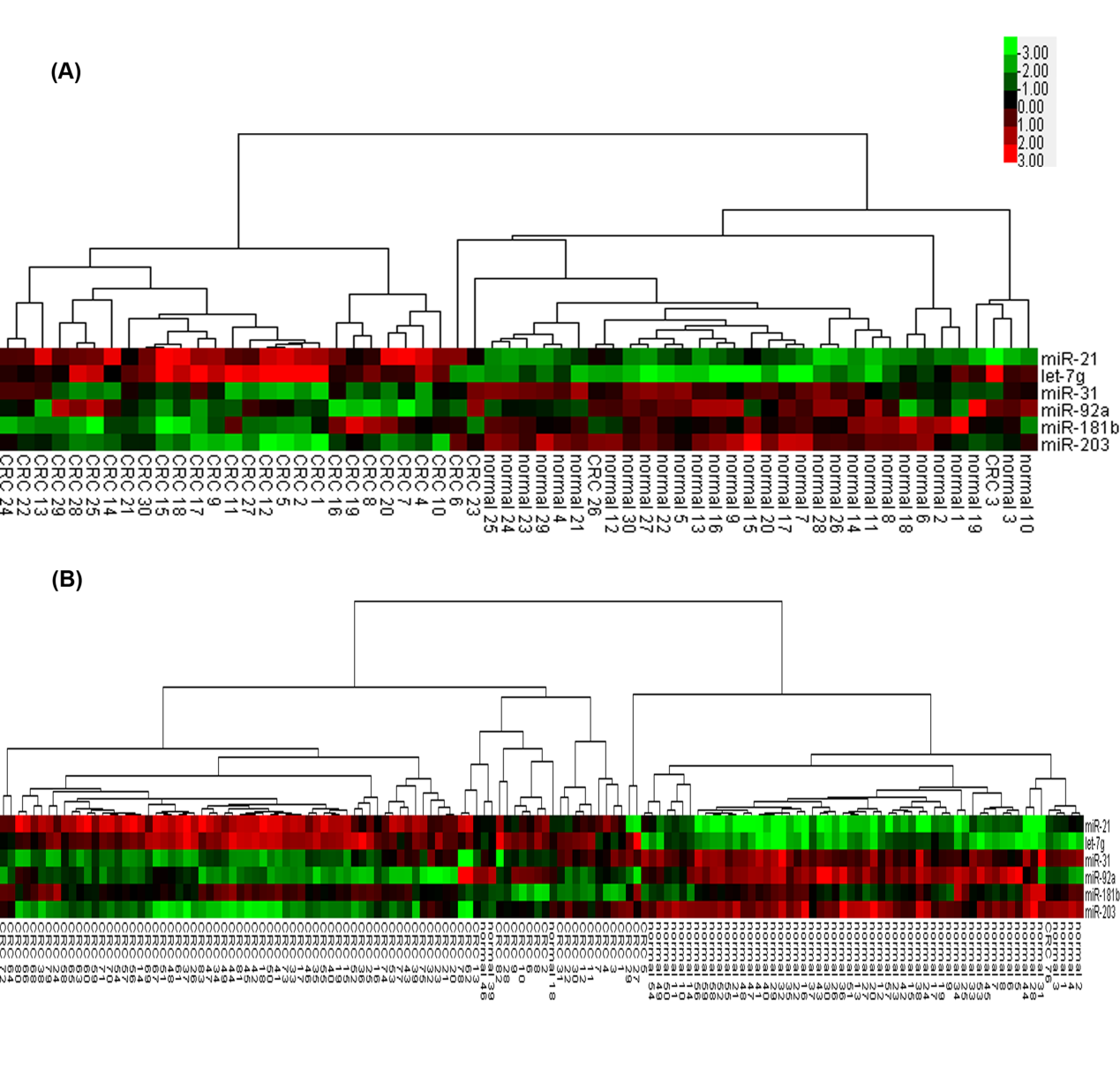


**Figure S2. Dendrogram of the unsupervised clustering results.** The dendrogram indicates a clear separation of the CRC samples from the control samples based on the six-miRNA signature in both the training set (A) and the validation set (B)
