# Supplementary material for: Identification of a Circulating MicroRNA Signature for Colorectal Cancer Detection
Source: PLoS One. 2014 Apr 7;9(4):e87451. doi: 10.1371/journal.pone.0087451 (PMC3977854; doi:10.1371/journal.pone.0087451)
Supplement: Table S1 — Differentially-expressed miRNAs in stage I/II CRC serum samples compared with in control serum samples. The normalized miRNAs expression levels are presented as mean ± SD. (DOCX) [file pone.0087451.s003.docx]

**Table S1. Differentially-expressed miRNAs in stage I/II CRC serum samples compared with in control serum samples.** The normalized miRNAs expression levels are presented as mean ± SD

| miRNA | Control  n=89 | Stage I/II CRC  n=62 | Average fold  change | p-value |
| --- | --- | --- | --- | --- |
| miR-21 | 1.40±1.31 | 6.51±3.59 | 4.65 | p<0.0001 |
| Let-7g | 0.98±0.78 | 5.43±8.34 | 5.54 | p<0.0001 |
| miR-31 | 1.2±1.29 | 0.31±0.49 | 0.25 | p<0.0001 |
| miR-92a | 1.87±2.21 | 0.48±1.17 | 0.26 | p<0.0001 |
| miR-181b | 1.48±1.81 | 0.6±0.99 | 0.41 | p=0.001 |
| miR-203 | 1.31±1.36 | 0.26±0.54 | 0.20 | p<0.0001 |
