# Supplementary material for: Identification of a Circulating MicroRNA Signature for Colorectal Cancer Detection
Source: PLoS One. 2014 Apr 7;9(4):e87451. doi: 10.1371/journal.pone.0087451 (PMC3977854; doi:10.1371/journal.pone.0087451)
Supplement: Table S2 — Sensitivity and specificity of the six-miRNA biomarker signature compared with the CEA and CA19-9 markers. (DOCX) [file pone.0087451.s004.docx]

**Table S2. Sensitivity and specificity of the six-miRNA biomarker signature compared with the CEA and CA19-9 markers.**

|  | Controls | CRC cases | Sensitivity | Specificity |
| --- | --- | --- | --- | --- |
| Pathological diagnosis | 89 | 113 | 100% | 100% |
| Serum six-miRNA biomarkers | 81 | 105 | 93% | 91% |
| CEA | 84 | 40 | 35% | 94% |
| CA19-9 | 86 | 26 | 23% | 97% |
